# Updates on Novel Non-Replacement Drugs for Hemophilia

**DOI:** 10.3390/ph15101183

**Published:** 2022-09-23

**Authors:** Roberta Gualtierotti, Samantha Pasca, Alessandro Ciavarella, Sara Arcudi, Andrea Giachi, Isabella Garagiola, Chiara Suffritti, Simona Maria Siboni, Flora Peyvandi

**Affiliations:** 1 Fondazione IRCCS Ca’ Granda, Ospedale Maggiore Policlinico di Milano, Angelo Bianchi Bonomi Hemophilia and Thrombosis Center, 20122 Milan, Italy; 2 Department of Pathophysiology and Transplantation, Università degli Studi di Milano, 20122 Milan, Italy; 3 Biomedical Sciences Department (DSB)/Medicine Department (DIMED) Padua University Hospital, 35131 Padua, Italy; 4 Department of Biomedical Sciences for Health, Università degli Studi di Milano, 20133 Milan, Italy

**Keywords:** hemophilia A, hemophilia B, nonreplacement therapy

## Abstract

Over the last decade, the world of hemophilia has experienced an unprecedented therapeutic advance, thanks to the progress in bioengineering technologies, leading to the introduction of drugs with novel mechanisms of action based on restoring thrombin generation or coagulation factor VIII mimicking. Apart from the bispecific monoclonal antibody emicizumab, already approved for patients with severe hemophilia A with and without inhibitors, novel non-replacement drugs designed to reduce the treatment burden of patients with hemophilia A or B with or without inhibitors are undergoing evaluation in clinical trials. Thanks to their innovative mechanism of action and subcutaneous administration, these drugs promise to provide effective bleeding protection together with improved adherence and improve health-related quality of life for patients with hemophilia. On the other hand, rare thromboembolic events have been reported with some of these drugs and warrant continuous post-marketing surveillance and investigation of predisposing factors, although the overall safety profile of most of these drugs is good. Finally, new challenges need to be faced in the clinical and laboratory monitoring of the hemostatic status in patients treated with these innovative therapies. In this review, we provide an update on the available data on novel non-replacement drugs currently undergoing evaluation in clinical trials for patients with hemophilia.

## 1. Introduction

Hemophilia A and B are rare bleeding disorders caused by a complete or partial deficiency in coagulation factors VIII (FVIII) or IX (FIX), respectively [1]. Children and adults with severe hemophilia (i.e., FVIII or FIX < 1 U/dL), if not adequately treated with a prophylactic infusion of the missing factor, may experience spontaneous musculoskeletal bleeding, accounting for 80% of overall bleeding events, although intracranial hemorrhage and other life-threatening bleeding events may also occur [2].

Over the last decades, exciting changes in the scenario of hemophilia treatment have occurred thanks to unprecedented progress in the pharmacological armamentarium available for these patients [3]. Despite these advances, significant treatment burdens and unmet needs still exist for patients with hemophilia. Most notably, prophylaxis with replacement drugs requires regular intravenous infusions, often affecting the patient’s health-related quality of life and patient adherence, which leads to the development of neutralizing alloantibodies (inhibitors) in approximately 20% of patients with hemophilia A and 2% of patients with hemophilia B, with subsequent complete or partial inactivation of replacement therapy [2,4,5]. For years, bypassing agents have been the only possible treatment for acute bleeding in these patients, although these drugs do not provide optimal bleeding protection and are burdened with high costs. Inhibitor eradication can be achieved with immune tolerance induction, i.e., the process of administering repeated regular high-dose infusions of the missing factor against which patients develop inhibitors with the aim of inducing tolerance. This process requires the presence of a central venous access, very frequent factor infusions, and is also burdened with high costs [2]. The licensing of emicizumab, a humanized anti-FIXa/FX bispecific antibody that acts as an FVIII-mimicking agent, has revolutionized the management of patients with hemophilia A with inhibitors, thanks to its mechanism of action independent of FVIII. In addition, the convenience of subcutaneous administration and long half-life (allowing weekly, fortnightly, or monthly administration) have led to the use of emicizumab in patients without inhibitors, resulting in increased adherence and improvement in health-related quality of life, in particular in patients with difficult venous access [6,7,8]. On the other hand, novel treatment options for patients with hemophilia B with inhibitors are currently not available, although particularly needed, as anaphylactic or severe allergic reactions to the infused FIX occur in approximately 50% of such patients, with many exhibiting a poor clinical response to immune tolerance induction [2,9,10].

**Figure 1:**
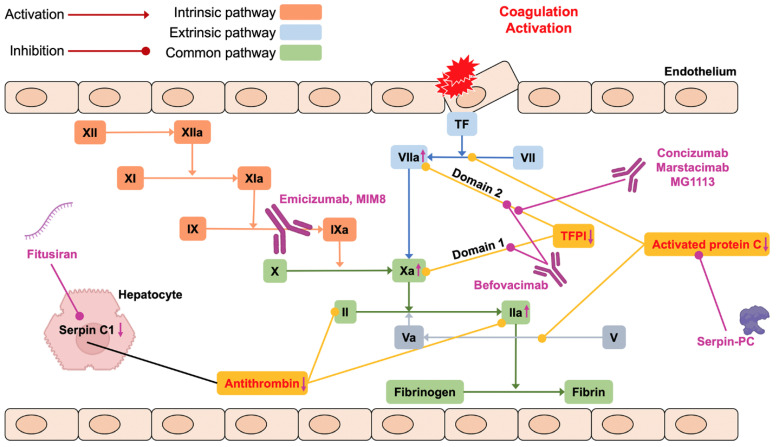
The mechanism of action of novel non-replacement treatments. PC = protein C; TF = tissue factor, TFPI = TF pathway inhibitor.

Novel non-replacement drugs have been designed and studied in order to provide an alternative to conventional replacement treatment or bypassing agents in patients with hemophilia A and B with or without inhibitors. The mechanism of action of these drugs is based on the knowledge that normal hemostasis depends on a delicate balance between natural procoagulants and anticoagulants (Figure 1). To date, two main approaches have been used: the first is the increase in defective thrombin formation through the inhibition of the naturally occurring anticoagulants, namely antithrombin, activated protein C, and tissue factor (TF) pathway inhibitor (TFPI) to rebalance the hemostatic system, an approach that can be applied to both hemophilia A and B; the second is a novel FVIII-activity mimicking agent, Mim8, which has been developed for patients with hemophilia A (Figure 1). These nonfactor therapies provide treatment for patients with severe clinical manifestations and limited treatment options, and improve adherence and health-related quality of life due to convenient subcutaneous administration. In contrast to replacement therapy (characterized by peak and trough plasma levels), which can be monitored by means of pharmacokinetic (PK) curves and is characterized by peaks and trough levels, non-replacement drugs, with their novel mechanism of action, reach stable plasma levels at a steady state, thus, changing the required type of laboratory monitoring to ensure efficacy and safety [11]. Finally, as hemostasis and coagulation are fine-tuned and very complex systems, an excess in thrombin generation may lead to thromboembolic events, which have been reported, although rarely [12].

With this narrative review, we hope to provide the reader with the latest updates on the novel non-replacement treatments for hemophilia A and B currently undergoing evaluation in clinical trials. The present review will not discuss studies with already approved drugs, such as emicizumab, nor other available treatment options with different mechanisms of action, such as gene and cell therapy.

## 2. Novel Non-Replacement Drugs

### 2.1. Fitusiran

The inhibition of the main natural anticoagulant antithrombin, which acts by inactivating activated factor X (FX) and thrombin, is an interesting therapeutic approach in the frame of the upcoming drugs for hemophilia A and B. Data on the influence of natural anticoagulant levels on the bleeding phenotype of patients with hemophilia was first reported in the literature more than ten years ago in a study demonstrating the association between thrombophilia abnormalities and a milder phenotype in patients with severe hemophilia A [13]. In a subsequent study, a murine model of hemophilia A with reduced plasma levels of antithrombin showed a restored coagulation characterized by increased levels of thrombin generation [14]. In this study, the inhibition of the synthesis of antithrombin by hepatocytes was achieved by exploiting the process of RNA interference (RNAi), a natural mechanism of gene silencing occurring in plants and mammals. Fitusiran is an investigational small interfering RNA (siRNA) that suppresses the synthesis of antithrombin in hepatocytes, thus, restoring sufficient hemostasis in patients with hemophilia A or B. Fitusiran is currently undergoing evaluation in registration studies in patients with or without inhibitors as prophylaxis administered subcutaneously every other month or once a month.

In phase 1, a dose-escalation study whose results were published in 2017, four healthy volunteers and 25 patients with severe or moderate hemophilia A or B without inhibitors received fitusiran or placebo [15]. A single dose of 0.03 mg/kg was administered to the healthy participants, while patients with hemophilia received three injections of fitusiran either once weekly (0.015, 0.045, or 0.075 mg/kg) or once monthly (0.225, 0.45, 0.9, or 1.8 mg/kg, or at a fixed dose of 80 mg). A sustained reduction in plasma levels of antithrombin was observed both after a single dose and with weekly regimens. Therefore, a monthly interval between doses was adopted, demonstrating a stable dose-dependent reduction in antithrombin of 70–89% from baseline and increased thrombin generation (ClinicalTrials.gov identifier: NCT02035605) [15]. Following these results, monthly fixed doses of fitusiran 50 and 80 mg were chosen for phase 2, open-label extension (OLE) study including 34 patients (15 with inhibitors and 19 without inhibitors) (NCT02554773) [16]. During the phase 2 study, a reduction in baseline levels of antithrombin between 85% and 72% was associated with an overall median annualized bleeding rate (ABR) of 0.84 and no clinically relevant complication occurred. Mild adverse drug reactions included asymptomatic increases in alanine transferase (ALT; ≥ 3x upper limit; 33% of patients, all positive for hepatitis C antibodies), injection-site reactions (18%), abdominal pain, diarrhea, and headache (9%). Of note, acute bleeding events were managed without complications in noninhibitor patients with doses of FVIII (mean 17.0 [range 5.0–31.0] IU/kg for a mean of 1.1 [1.0–2.0] doses) or factor IX (FIX) (a mean of 18.0 [9.0–27.0] IU/kg for a mean of 3.9 [1.0–8.0] doses) lower than the recommended dose for breakthrough bleeding management [2]. Similarly, patients with inhibitors received lower doses of activated prothrombin complex concentrate (aPCC) (mean of 27.0 [14.0–37.0] U/kg for a mean of 1.5 [1.0–3.0] doses) or recombinant FVIIa (rFVIIa) (mean of 56.0 [37.0–62.0] µg/kg for a mean of 2.7 [2.0–3.0] doses) than previously administered before starting fitusiran [17].

In September 2017, the occurrence of a fatal case of cerebral thrombosis in a young patient without inhibitors participating in the phase 2 extension study led to a temporary suspension of clinical trials involving the drug (Table 1). The patient, who had been treated for exercise-induced hip pain with three doses of FVIII (31.0–46.0 IU/kg), developed a severe headache on the last day of treatment and was diagnosed by computed tomography (CT) scan with subarachnoid hemorrhage. Thus, the patient received additional treatment with FVIII, and after a 14-day hospitalization period, he died from cerebral edema. A CT scan post-hoc review demonstrated that the event was a sinus vein thrombosis rather than a subarachnoid hemorrhage [18]. In November 2017, clinical trials were resumed following the availability of an agreement between the manufacturer and the US Food & Drug Administration on a risk mitigation strategy proposed to the investigators. In addition, the protocol of the ongoing phase 3 study included guidelines and patient education about fitusiran and the use of concomitant drugs (e.g., FVIII concentrates or bypassing agents) aimed to contain thromboembolic risk.

Due to further reports of nonfatal thrombotic events, all fitusiran investigations were paused in October 2020. In particular, as of November 2020, 259 patients had been treated with at least one dose of fitusiran in the clinical trial program and five thrombotic events had been reported: the already mentioned case of cerebral venous sinus thrombosis; a cerebral vascular accident; a cerebral infarct; a spinal vascular disorder; and a case of atrial thrombosis (Table 1). Four of the five events happened in patients with hemophilia A, three without inhibitors. The fifth event occurred in a patient with hemophilia B with inhibitors. This led to a revision of the study protocol on fitusiran dose and regimen as risk mitigation for vascular thrombosis that was presented at the 2021 EAHAD Congress. The revised dosing plan mandates that patients start with a 50 mg dose of fitusiran every other month. Fitusiran dosing was discontinued in patients with two measurements of antithrombin levels < 15%. Patients at a steady state who had two antithrombin level measurements > 35% were permitted to escalate to a higher dose. The two escalating dosing regimens were either 50 mg or 80 mg monthly, depending on the antithrombin levels.

The safety and efficacy of fitusiran have been evaluated in patients with hemophilia A and B with (ATLAS-INH, NCT03417102) or without (ATLAS-A/B, NCT03417245) inhibitors, and in patients receiving prophylactic therapy (ATLAS-PPX, NCT03549871). Preliminary reports of the results of ATLAS-INH and ATLAS-A/B were presented at the annual meeting of the American Society of Hematology (ASH) in December 2021 [19,20]. In both patients with and without inhibitors, treatment with fitusiran was associated with a nearly 90% reduction in ABR.

The ATLAS-OLE is a study that aims to evaluate the long-term safety and efficacy of fitusiran in patients with hemophilia A or B, with or without antibodies to FVIII or FIX (NCT03754790), which is still recruiting. In addition, phase 3 studies are currently ongoing in adolescents, and a dose-confirmation study has been initiated in children as well (ATLAS-PEDS, NCT03974113).

### 2.2. SerpinPC

Another treatment strategy is based on the interference with activated protein C (aPC). The thrombin-thrombomodulin complex activates PC to produce aPC, which inactivates FVIIIa and FVa in association with its cofactor protein S, thereby inhibiting further thrombin formation. This leads to the inactivation of the prothrombinase and tenase complex activities [21].

The inhibition of aPC, therefore, would restore the hemostatic balance in patients with hemophilia by prolonging thrombin formation. SerpinPC is an aPC inhibitor currently being evaluated in phase 2a, a proof-of-concept trial in patients with severe hemophilia A and B (AP-0101, NCT04073498) [22]. Polderdijk et al. designed a modified serpin on the scaffold of the Pittsburgh variant of α1-antitrypsin, in which the native P2-P1′ sequence Pro-Arg-Ser was replaced by the Lys-Arg-Lys sequence. This modified serpin specifically binds aPC with a covalent bond, without binding a nonactivated PC, and does not inhibit thrombin [23]. The preserved function of secondary aPC possibly accounts for the absence of thrombotic events reported in clinical trials so far.

The safety and efficacy of this modified serpin are currently being evaluated in a phase ½ clinical trial in healthy volunteers and in patients with severe hemophilia. Interim analysis was performed on 23 patients with severe hemophilia without inhibitors treated with on-demand therapy (19 patients with hemophilia A and 4 patients with hemophilia B). The drug was administered as a subcutaneous injection every four weeks and three doses were evaluated: 0.3, 0.6, and 1.2 mg/kg. The primary outcome was assessed after a 24-week follow-up. A median 88% reduction in all bleeds (from 36.0 to 4.4) was observed in the last 12 weeks of treatment (prespecified primary assessment period) compared to the overall ABR prospectively measured over the pre-exposure observation period with the highest dose. Self-reported spontaneous joint ABR was reduced by 94% (from 21.1 to 2.2) in the highest dose cohort. Similar results were observed in hemophilia A and B. The median number of target joints (i.e., joints with >3 bleeds within six months) was zero at the end of the study compared with a pre-exposure baseline of 2.5. In particular, all subjects had target joints at the beginning of the study, and 15 subjects had zero target joints at the end of the study. Safety was good: no venous thromboembolic events or other severe adverse effects were reported. No sustained elevation in D-dimer levels was reported throughout the time of follow-up. A patient with a history of a skin disorder experienced an injection site reaction, leading to the discontinuation of the treatment. Two patients developed antidrug antibodies (ADA), apparently not affecting the drug’s effectiveness [22]. All 22 patients who completed the phase 2a part of the study were considered eligible for the 48-week open-label extension part of the study, in which a single fixed 60 mg subcutaneous dose of serpinPC will be administered every four weeks over 48 weeks (13 doses total).

### 2.3. Anti-TFPI

The role played by the tissue factor/activated factor VII (TF/FVIIa) complex in the coagulation cascade activation was first highlighted by Roberts et al. in their in vitro model, which mimics the in vivo condition [24]. This pathway initiates coagulation by activating both FIX and FX. The result of this double activation is the conversion of prothrombin into thrombin, with the final formation of the fibrin clot. The TFPI is a serine protease inhibitor described by Wood et al. and by Broze and Girard [25,26] that turns off this reaction, with an irreversible process that requires the presence of calcium, enhanced by the action of protein S, thus, allowing proper hemostasis to be maintained. The TFPI gene was first cloned and characterized in 1988 [27]. It is located on the long arm of chromosome 2 (q32), contains 10 exons and 9 introns, and measures approximately 90 Kb. It is present in three different isoforms: (1) TFPIα, the isoform most present in the plasma, but purified also in the conditioned media of HepG2 cells, containing an acidic aminoterminal (N-) region followed by three tandem Kunitz domains encoded by exons 4-6-9 and the intermediate peptides that are allocated among Kunitz domains encoded by exons 5 and 7, and by a carboxyterminal (C) region; (2) the TFPIβ initially discovered in mice, but also found in the ECV304 human cells of bladder cancer; and (3) the TFPIδ more expressed in the liver. TFPI has a molecular weight between 34 and 41 kDa and circulates in plasma bound to lipoproteins; its mean concentration is usually approximately 70 ng/mL. TFPIα can be proteolytically degraded by a different proteinase, or by FXa if its molar quantity exceeds that of the TFPIα itself. In vitro models showed that the plasma concentrations of TFPIα may also be reduced in people with FV or protein S deficiencies, and presumably it could also be reduced in those with FVIII deficiency. The activity of the TFPIα is concentrated at the level of the Kunitz domains: the first inhibits the TF/FVIIa complex, the second inhibits FXa, while the third is only involved in binding to low-density lipoprotein and heparins and has no known inhibitory activity. The C-terminal region permits the simultaneous interaction of FXa and TFPIα to the phospholipid surface, and it is also involved in FXa inhibition. In vitro models showed that the TFPI molecules lacking from this region had a reduced anti-Xa and anticoagulant effect. All these inhibitory actions of the TFPI, which consequently led to a drastic reduction in thrombin generation, could help explain the excessive bleeding in patients with hemophilia. The mechanism of action of TFPI is shown in Figure 1.

The first anti-TFPI that has been tested was BAX499 (ARC19499), binding Kunitz 1 and three domains. The drug has not been included in the review because the phase 1 study (NCT01191372) was stopped due to an increase in the number of bleeding events, and therefore, it has not reached phase 2 and 3 studies [28,29].

#### 2.3.1. Concizumab

Concizumab is a monoclonal, humanized IgG4 antibody specifically binding to the Kunitz-2 domain. Preclinical studies in vitro or in animal models have described its action in restoring thrombin generation and favoring the onset of a procoagulant action [30,31,32,33].

The Explorer program was then launched to confirm these data and to allow subsequent use in patients with hemophilia. Explorer 1 was the first trial belonging to this program performed on humans (NCT01228669) [34,35]. Escalating single doses of concizumab (intravenously or subcutaneously) were administered to healthy volunteers and patients with hemophilia A or B. This was a phase 1, multicenter, randomized, double-blind, placebo-controlled, single-dose, and dose-escalation trial. The enrolled participants were subsequently randomized (3:1) to receive a single dose of concizumab (three subjects) or a placebo (one subject). Fifty-two participants were included in Explorer 1 trial, 28 were healthy volunteers, while 24 were patients with hemophilia (21 with hemophilia A and 3 with hemophilia B). The tapered dosage was first tested in healthy volunteers and then in patients with hemophilia. The primary endpoint of this study was safety, assessed at intervals up to 43 days after concizumab administration. Throughout the trial, overall, 76 adverse events were reported, 75% of them were mild, none of which were serious. Nineteen adverse events occurred in the placebo group. No correlation was found among different doses of concizumab and the onset of adverse events was found. PK and pharmacodynamics (PD) were also assessed throughout the whole study. Concizumab exhibited a nonlinear PK with no difference between healthy volunteers and patients with hemophilia. PD analyses showed that unbound TFPI plasma concentration profiles were inversely related to PK ones, with unbound TFPI remaining low for more than 14 days after the highest concizumab dose was reached. Twenty-four bleeds were reported in a total of 14 patients with hemophilia (five placebo; nine concizumab), while only one bleed occurred in a healthy volunteer in the concizumab arm. Only one bleed was further described in a subject presenting high concizumab levels and a low free (unbound) TFPI concentration in plasma. No differences were found between intravenously or subcutaneously administration. This study, therefore, demonstrated that concizumab can be considered safe and that the route of administration does not change its characteristics.

The Explorer 2 trial (NCT01631942) had safety as a primary endpoint as well, together with the PK/PD assessment after the subcutaneous administration of concizumab in healthy male subjects and patients with hemophilia. The Explorer program continued with Explorer 3 trial, another study on the safety of concizumab in patients with hemophilia (NCT02490787) [36]. This multicenter, phase 1b, placebo-controlled trial investigated five different escalating doses of the experimental drug in many groups. The assessed endpoints were safety, PK/PD, and subcutaneous immunogenicity of concizumab at different established doses. Overall, 24 participants were enrolled in this study, equally divided into three different dose groups (0.25-0.5-0.8 mg/kg) with subcutaneous administration every four days. Six patients from each group were included in the concizumab arm, two in the placebo one. A total of 56 adverse events occurred in 19 patients, 54 of them were mild and two were moderate. The number of adverse events was not correlated with concizumab dose, as found in Explorer 2 trial. Ninety-one bleeds were reported, 28 of which occurred in five patients in the placebo arm. Almost all bleeds were mild, except for a spontaneous hemarthrosis and a traumatic muscle bleeding occurring in the concizumab arm (0.5 mg/kg) and in the placebo arm, respectively.

Explorer 4 (NCT03196284) and Explorer 5 (NCT03196297) trials were both phase 2 studies designed to assess the efficacy and safety of subcutaneous concizumab administration in patients with hemophilia A or patients with hemophilia B with inhibitors (Explorer 4) and in severe patients with hemophilia A without inhibitors (Explorer 5), and to establish the optimal dose of experimental drug to be used in the subsequent phase 3 studies [37,38]. Data on the long-term efficacy and safety of concizumab prophylaxis in these two trials has been recently published, further supporting the use of concizumab as a daily prophylactic treatment in all hemophilia subjects [39]. Both Explorer 4 and Explorer 5 trials included main (≥24 weeks) and extension (52–102 weeks) studies, with patients receiving 0.15 mg/kg of concizumab with a dose escalation of 0.20 or 0.25 mg/kg if they experienced at least three treated spontaneous bleeding episodes within 12 weeks. Overall, 36 patients with hemophilia A, 15 patients with hemophilia A with inhibitors, and 10 patients with hemophilia B with inhibitors were enrolled in these two studies. In Explorer 4 trial, three patients withdrew before completing the extension study: one due to lack of efficacy on the last dose level; one due to suspicion of no therapeutic effect due to normal TFPI levels in the presence of ADA; and one due to consent withdrawal. In Explorer 5, four patients withdrew during the main part and three during the extension part: one with no stated reason, and two due to protocol-defined lack of efficacy at last dose level. A total of 12 out of the 25 patients remained on a dose of 0.15 mg/kg of concizumab (seven patients with hemophilia A with inhibitors and five patients with hemophilia B with inhibitors) during the extension part of Explorer 4, and 15 out of 36 in the extension part of Explorer 5. These results have allowed the researchers to establish the optimal dose of concizumab to be used in subsequent phase 3 studies, which will include an initial loading dose on the first day, in order to reach the steady state, followed by a maintenance dose with the highest dose used in the phase 2 trials (0.25 mg/kg).

Estimated ABR during the main and extension studies at the last dose levels were 4.8 (1.8 spontaneous ABR) in Explorer 4, and 6.4 (2.1 spontaneous ABR) in Explorer 5. Concizumab was generally well tolerated in patients with and without inhibitors, with no serious adverse events leading to withdrawal. In particular, no thromboembolic events or deaths were reported. The majority of injection site reactions reported were bruising, hemorrhages, and hematomas, mainly occurring in patients on the concizumab dose of 0.15 mg/kg. ADA developed in six and nine patients during the main and extension parts of Explorer 4 and in Explorer 5, respectively. In all patients except one, ADA were at low titer, whereas one patient from Explorer 4 with initially low-titer ADA developed a high titer with neutralizing activity in vitro. The patient continued to receive concizumab, despite free TFPI restoration, reporting two bleeding events over a 7-month period and was eventually withdrawn due to suspicion of no therapeutic effect with restoration of free TFPI.

In March 2020 nonfatal thrombotic events were reported in three different participants (Table 1), all with distinct thrombotic risk factors [40]. These observations suggest the need to pay close attention to subjects at greater risk of thromboembolic events, such as the elderly with underlying cardiovascular problems, or in the case of association with other hemostatic drugs. Subsequently, the 13 patients recruited in Explorer 5 who were still receiving concizumab dosing were asked to discontinue treatment and attend the end-of-treatment visit, with the last patients receiving a concizumab dose on 18 March 2020. The pivotal studies Explorer 6 (NCT03741881), Explorer 7 (NCT04083781), and Explorer 8 (NCT04082429) were temporarily stopped and restarted a few months later, but with a new reduced dosing regimen of the experimental drug to mitigate the thromboembolic risk, as recently presented by Chowdary et al. and Astermark et al. to the “15th Annual Congress European Association of Haemophilia and Allied Disorders (EAHAD)” [40,41].

The Explorer program is still ongoing, and the phase 3 trial results are not published yet, only few abstracts related to safety are currently available.

#### 2.3.2. Marstacimab

Marsticimab (or PF-06741086), is a human IgG1 monoclonal antibody drawn to target the complex TF/FVIIa in the extrinsic pathway of the coagulation cascade by binding to the Kunitz 2 domain of the human TFPI (17). Marstacimab is a drug in development as a potential prophylactic subcutaneous treatment to prevent bleeding episodes in hemophilia A and B patients with and without inhibitors. The phase 1 trial enrolled 42 healthy male volunteers, and was designed to evaluate safety, tolerability, and PK/PD of study drug at doses ranging from 30 to 400 mg administered both intravenously and subcutaneously. The subjects treated with a low dose of marstacimab were followed for 42 days after the first administration, while the subjects treated with high doses were followed for 84 days. The experimental drug was found to be safe at any dosage, which, therefore, allowed it to continue with the subsequent phases of the study [42].

The phase 1b/2 multicentre trial (NCT02974855) evaluated efficacy, safety, and PK/PD of marstacimab, in patients with hemophilia A and B, with or without inhibitors. Twenty-six participants were enrolled in this study and treated with the experimental drug at four different doses based on the patients’ disease: 300 mg was administered to patients without inhibitors; while 150, 300, and 450 mg were administered to patients with inhibitors. All subjects were followed for 113 days after the first administration. Four severe adverse events were reported, none related to the study drugs. No significant changes in the laboratory nor in the thromboses were reported. Three subjects developed ADA, but none of them had a neutralizing effect. The efficacy endpoint was assessed evaluating the bleeding that occurred in participants six months before and six months after trial initiation. Overall, the reduction in bleeding rate averaged 80–96% [43,44]. The phase 3 trials are still ongoing.

#### 2.3.3. Other Anti-TFPI Antibodies

Two other anti-TFPIs developed for the treatment of patients with hemophilia deserve a brief mention: befovacimab (BAY 1093884) and MG1113 [33]. Befovacimab is a human IgG2 monoclonal antibody created to bind both to the Kunitz domain 1 and 2. The results of the phase 1, multicenter, open-label trial, performed in patients with hemophilia A and patients with hemophilia B, with or without inhibitors were presented in 2018 by Chowdary et al. to the 60th Congress of American Society of Hematology (ASH) [45]. Safety and PK/PD were assessed after a single intravenous (0.3 and 1.0 mg/kg) and a single subcutaneous (1.0, 3.0, and 6.0 mg/kg) dose administration. No adverse events were observed, while the PK/PD profiles were similar in hemophilia A and B, with or without inhibitors. With this background, a phase 2 trial (NCT03597022) was then started. This is a multicenter, dose-escalating study evaluating the safety of the experimental drug in patients with hemophilia A and patients with hemophilia B, with or without inhibitors, divided into three dose groups (100, 225, and 400 mg). Overall, 24 patients were enrolled, and the preliminary results showed a reduction in bleeding events in the high and intermediate dose group, but not in the lower-dose one. Three patients, two in the intermediate arm and one in the high-dose arm, experienced central nervous thromboses (Table 1). These events occurred in the absence of the differentiating PK/PD characteristics in patients who experienced thromboses to predict the onset of adverse events during treatment. This led to the definitive closure of the study program on befovacimab [46]. The etiological mechanism leading to the development of thrombosis could bind to the Kunitz 1 and 2 domains, characteristic of befovacimab. Alternatively, the different distribution of TFPI in endothelial cells of different organs may explain the central nervous system localization of the thrombotic events [12].

MG1113 is a humanized IgG4 monoclonal antibody that binds the Kunitz 2 domain with a nonlinear PK after both intravenously and subcutaneously administration, such as concizumab. The phase 1 trial (NCT03855696) is still ongoing, while the results of the in vitro and in vivo studies in animal models showed a restoration of thrombin generation and a reduction in bleeds [47,48].

### 2.4. Mim8

Mim8 (NNC0365-3769) is a novel, next generation, FVIII-mimetic bispecific antibody for subcutaneous prophylactic treatment for patients affected with hemophilia A, with and without inhibitors. Mim8 was developed by means of the Duobody^®^ platform [49], in order to screen anti-FIXa and anti-FX antibodies. In total, more than 30,000 bispecific antibodies were analyzed, and subsequently an optimization process of the anti-FIX arm was performed with the aim to improve Mim8 characteristics [50]. The final product showed low FIX and FX binding in solution, strengthened activation at the membrane site, low immunogenicity, and low viscosity [51]. Although sharing the bridging FIXa to FX function with emicizumab, Mim8 has a different molecular structure [51]. In particular, the anti-FIXa arm of Mim8 recognizes different epitopes than the emicizumab arm [50]. This difference was also confirmed by the findings of our group, showing that anti-emicizumab antibodies do not react with Mim8 via an in-house in vitro assay [30]. Mim8 exhibits a 15-fold greater ability to normalize thrombin generation in vitro compared to emicizumab [50]. Currently, phase 1 and phase 2 clinical trials are ongoing, with the aim to assess the efficacy and safety of Mim8 in vivo (NCT05127473; NCT04204408). Promising results were obtained with animal models, both in monkeys and mice [51,52]. Two phase 3 trials are currently ongoing in adults and adolescents with hemophilia A, with or without inhibitors (NCT05053139) and in children with hemophilia A, with or without inhibitors (NCT05306418), both recruiting.

## 3. Safety Issues and Effects on Coagulation Assays of Novel Non-Replacement Therapies

Thrombotic events have been described with fitusiran, befovacimab and concizumab (Table 1) [12,39]. These events underscore the subtle line separating hemostasis activation and thrombosis. In particular, the potential thrombotic risk in patients with hemophilia receiving novel nonfactor therapy together with replacement therapies or bypassing agents for acute bleeds is of great concern [12]. The risk of excessive thrombin generation with novel non-replacement drugs warrants continuous monitoring of thrombotic risk with these drugs. In order to prevent thrombotic events with novel non-replacement drugs, the contribution of individual risk factors (e.g., family or personal history of thrombosis, increasing age, obesity, hypertension, smoking habit, dyslipidemia, diabetes mellitus, cardiovascular diseases, cancer, and inflammatory diseases) should be considered in clinical decision-making regarding possible switching to these drugs. The usefulness of screening for inherited or acquired thrombophilia, such as factor FV Leiden, prothrombin gene variant, protein C, protein S, antithrombin deficiency, and lupus anticoagulant, in candidates for non-replacement is still debated. In patients with thrombotic or cardiovascular risk factors careful risk-benefit evaluation should be performed and the pros and cons of the novel treatment should be discussed thoroughly with the patient. Long-term monitoring for thrombotic events and patient education to recognize and report signs and symptoms of arterial or venous thrombosis should be encouraged as well. Apart from the clinical monitoring of signs and symptoms of arterial, venous thromboembolic events, and microangiopathies, the coagulation state of the patients could be monitored by laboratory tests, although no recommendations have been published for the laboratory monitoring non-replacement drugs, or for conventional bypassing agents.

Based on their mechanisms of action, the measurement of global coagulation by means of assays, such as thrombin generation assays or whole blood thromboelastometry seems to be the most appropriate approach for these drugs. Thrombin generation assays evaluate the balance between the generation of thrombin resulting from the action of the procoagulants and its decay resulting from the action of the anticoagulants driver. During the thrombin generation assay, the coagulation of the test plasma (platelet poor or platelet rich) is activated by small amounts of TF and phospholipids, and the reaction of thrombin generation is continuously monitored by means of a thrombin-specific fluorogenic substrate. The main parameters resulting from the employment of this assay are the endogenous thrombin potential, defined as the net amount of thrombin that test plasma samples can generate on the basis of the relative strength of the pro- and anticoagulant drivers; the lag time, which is the time of the lag phase that follows the addition of the trigger until the initiation of thrombin generation; the thrombin peak expressed in nmol/L; the time it takes to peak, expressed in minutes; the velocity index, defined as the peak height/(time to peak—lag time) ratio; and the area under the curve, which is the actual endogenous thrombin potential (nmol/L of thrombin x min) [53]. However, the test does not take into account other hemostatic mechanisms, such as platelet contribution, fibrinogen function or fibrinolysis. For these reasons, thromboelastography and rotational thromboelastometry (ROTEM) have been proposed as an alternative global coagulation assay [54]. Both measure the adhesiveness, elasticity and other physical properties of blood from a dynamic and global perspective, using a vertical pin held in the blood sample, contained within a cup or cuvette: in thromboelastography the cup oscillates clockwise and anticlockwise with the blood clots, whereas in the ROTEM, an oscillatory force is applied to the pin and the cuvette is held stationary [55]. Thromboelastography parameters are reaction times, which are the time interval between the start of coagulation and the point where a 2-mm amplitude is reached; the coagulation time, which is the time interval between the start of coagulation and the point where amplitude reaches 20mm; the alpha angle that shows the rate at which a solid clot is formed; the maximum amplitude (mm), i.e., the greatest diameter of the clot and a measure of clot elasticity; the maximum amplitude at 60 min (mm); the clot lysis index (%), that describes the amount of the clot that shows fibrinolysis in a set time (30 min); an indicator of how firm the clot is; the clot lysis, which measures the time interval between the maximum amplitude and zero amplitude, and is a marker of fibrin lysis activity [56]. ROTEM parameters are the clotting time, which are the time needed to start clot formation, usually until a 2-mm amplitude is reached; the clot formation time is the time needed to reach an amplitude of 20 mm; the angle alpha, describing the kinetics of clot formation; the maximum clot firmness is the maximum amplitude of the curve; amplitude at different timepoints and the clot lysis index at 30 and 60 min are described as well; finally, the maximum lysis represents the maximum degree of fibrinolysis detected during the measurement [57]. In vitro global coagulation assays show a very high variability from patient to patient and across laboratories and, therefore, they are not currently used for monitoring individual thrombotic risk. This is the main reason why they are not currently used as routine monitoring of individual thrombotic risk.

Fitusiran subcutaneous injection was shown to normalize ex vivo thrombin generation, with an association with the reduction in antithrombin activity [18]. In particular, in patients with ≥75% antithrombin reduction, peak thrombin generation values were within the low normal range of healthy volunteers [15,58]. In vitro data have shown an additive effect of antithrombin reduction and bypassing agents on thrombin generation in plasma from people with hemophilia [58]. Consistently, the median aPCC dose used to treat bleeding events in the phase 1 trial was 28.6 U/kg, representing approximately 50% of the usual dose of aPCC employed to manage breakthrough bleeding in patients with inhibitors [15]. Monthly subcutaneous administration of 50 mg or 80 mg fitusiran prophylaxis over a period of 48 months resulted in sustained lowering of antithrombin levels (a reduction in approximately 80% from baseline), leading to peak thrombin levels and an endogenous thrombin potential approaching the normal range seen in healthy volunteers [59].

Serpin PC was shown to restore the effect of soluble thrombomodulin in normal and factor-deficient human plasma in vitro in a dose-dependent manner [23]. In this experimental condition, the addition of thrombomodulin in vitro was needed to activate the protein C system, as protein C is activated from its precursor zymogen by thrombin bound to the cofactor thrombomodulin on the surface of endothelial cells, the addition of thrombomodulin in vitro in this experimental condition was needed to activate the protein C system.

The effect of concizumab on thrombin generation was investigated in healthy individuals receiving subcutaneous injections of concizumab and in vitro experiments of drug-spiked hemophilia plasma. Both showed a dose-dependent increase in thrombin generation [34,60]. Waters et al. studied a total of 22 participants, 18 of whom were patients with hemophilia, while the other four were healthy subjects whose blood samples were obtained from Explorer 2 trial [60]. The thrombin generation assay showed increased thrombin generation both in plasma samples obtained from patients with hemophilia after spiking with increasing concentrations of concizumab, and in plasma samples obtained from healthy volunteers receiving successive doses of experimental drug every other day, for 15 days thrombin generation increased. In data collected in Explorer 3 study, a strong correlation between concizumab concentration and peak thrombin generation potential was observed. In particular, concizumab >100 ng/mL restored thrombin generation potential within a normal range. A strong correlation between concizumab exposure and free TFPI was also observed, thus supporting free TFPI (ie, TFPI in plasma, unbound to concizumab) measurement as a useful biomarker for concizumab efficacy. Notably, fewer bleeds were observed with concizumab concentrations > 100 ng/mL and at free TFPI levels < 25% compared to baseline [61]. In another study, pooled hemophilia A plasma samples were spiked in vitro with concizumab alone or together with rFVIIa, aPCC or rFVIII, in a second set of experiment, rFVIIa, aPCC, rFVIII were added ex vivo to plasma from hemophilia A patients receiving concizumab prophylaxis; and finally in pooled hemophilia B plasma samples were spiked with concizumab alone or together with rFIX. Thrombin generation assay demonstrated that concizumab was able to increase thrombin peak in a concentration-dependent manner. The addition of rFVIIa, aPCC, rFVIII, or rFIX caused a further increase in thrombin peak with an additive effect and no strong synergistic effects. Drug-drug interaction was associated with up to 25% extra effect when combining concizumab with rFVIIa, APCC, rFVIII, or rFIX. In particular, the thrombin peak obtained with 0.5 IU/mL rFVIII or rFIX in the presence of concizumab was comparable to the thrombin peak observed with 1 IU/mL rFVIII or rFIX in the absence of concizumab [62]. Similarly, ROTEM analysis of hemophilia A-like blood obtained adding FVIII antibodies to citrate-stabilized normal whole blood spiked with concizumab resulted in concentration-dependent responses [63]. In particular, the thrombin peak obtained with 0.5 IU/mL rFVIII or rFIX in the presence of concizumab was comparable to the thrombin peak observed with 1 IU/mL rFVIII or rFIX in the absence of concizumab [62]. Similarly, ROTEM analysis of hemophilia A-like blood obtained adding FVIII antibodies to citrate-stabilized normal whole blood spiked with concizumab resulted in concentration-dependent responses [63].

Even for marstacimab evidence comes from in vitro spiking of hemophilic A or B or nonhemophilic plasma samples with the drug alone or in combination with rFVIIa or aPCC. Hemostatic activity was measured using the thrombin generation assay, showing that a combination of marstacimab at a 16.0 µg/mL dose plus rFVIIa 2.0 µg/mL or aPCC 1 UI/mL (all corresponding to plasma levels that could be achieved clinically after dosing) slightly increased peak thrombin levels compared with either agent alone. This increase was within the reported range for nonhemophilic plasma. In addition, this increase did not exceed levels observed in nonhemophilic plasma treated with marstacimab alone [64]. In another study, participant plasma samples spiked with marstacimab exhibited improvements in thrombin generation parameters, including reduced lagtime and increased peak thrombin concentrations. The assay was conducted also with platelet-rich plasma, and in this condition the effect of marstacimab on decreasing thrombin generation assay lagtime was more pronounced, possibly due to the assay conditions with the presence of platelet membrane phospholipids or to the contribution of platelet FV [65]. Similarly, marstacimab induced pro-coagulant responses in ROTEM parameters such as reduction in clotting times and increases in angle. Notably, in both ROTEM and thrombin generation assay, 100.0 nmol/L marstacimab, which is a clinically relevant concentration, showed effects comparable to ex vivo addition of 40% rFVIII or rFIX activity.

Mim8 shown to be able to restore thrombin generation using thrombin generation assay in hemophilia A plasma and to normalize blood clot formation using thrombelastography in whole blood samples from healthy volunteers spiked with anti-FVIII antibodies [50]. In addition, the effects of Mim8 and rFVIIa in thromboelastography were studied using blood from healthy donors created hemophilia A-like through the addition of anti-FVIII antibodies. Mim8 and rFVIIa had additive effects that were lower in magnitude than those observed with the combination of Mim8 and aPCC or with an emicizumab sequence identical analogue and aPCC [66]. As far as the interference of these drugs with one stage and chromogenic assays it is well known that emicizumab concentration can be measured by means of a modified one-stage FVIII clotting assay and that a bovine chromogenic assay, which is insensitive to emicizumab can be used to measure FVIII levels in plasma of patients with hemophilia without inhibitors [67]. As for the novel non-replacement drugs, evidence is scant. An aPTT-based assay appears feasible in the presence of both fitusiran and anti-TFPI based on the coagulation mechanisms of these agents. In addition, these molecules do not relevantly interfere in PT, INR and fibrinogen assays [68]. Preliminary data from in vitro studies suggest that chromogenic bovine FIXa/FX assays are insensitive to Mim8 and can be used to accurately measure additional recombinant FVIII therapy or FVIII inhibitors in patients treated with Mim8. Mim8 concentration can be accurately determined by a modified chromogenic assay [69].

Specific monitoring of fitusiran activity would be possible by measuring residual antithrombin levels, either via antigen assays or via thrombin- or FXa-based activity assays [70]. No specific assay is currently available to measure anti-TFPI antibody levels, thus free TFPI levels should be analyzed instead. Antigen levels of TFPI can be measured with established ELISA systems. However, it is unclear whether all therapeutic anti-TFPI antibodies actually reduce plasma TFPI levels or whether these monoclonal antibodies interfere in ELISA assays. Alternatively, specific activity assays to measure residual TFPI activity can be used. These include a diluted prothrombin time–based assay and TF–dependent chromogenic assays. It should be noted that plasma contains approximately 10–30% of all TFPI and therefore these assays will not retrieve a full comprehension of the amount of TFPI is inhibited at the vascular lining [11,71,72,73].

## 4. Discussion

Hemophilia care is currently experiencing a very active period of therapeutic advances thanks to unprecedented research progress.

Although novel non-replacement drugs treated in the present review are still undergoing evaluation in clinical trials, if licensed, they will facilitate prophylaxis in patients with hemophilia A and B, in particular, but not only in patients with inhibitors. Overall, these novel compounds are characterized by the capacity to improve thrombin generation in an FVIII-independent manner, by subcutaneous administration, no cross-reactivity with FVIII-inhibitors, and long half-lives permitting infrequent dosing in most cases, thus, improving adherence to treatment, and improving health-related quality of life of patients with hemophilia. In addition, besides their primary use for the prevention of bleeding episodes in patients with hemophilia and inhibitors, patients with rare bleeding disorders other than hemophilia may benefit from this novel approach. Actually, the off-label use of these novel non-replacement drugs in these groups of patients is currently being debated.

It should be noted that the introduction of these novel therapies will lead clinicians to face some important challenges. Despite the benefits of rebalancing hemostasis with novel non-replacement therapies, the potential risk of thrombosis still needs to be fully acknowledged. Rare reports of thromboembolic events during clinical trials have led pharmaceutical companies to change their study protocols and warrant continuous post-marketing surveillance. Currently, it is not possible to predict the differential response to one non-replacement product or another because the number of patients treated with these drugs in clinical trials is still limited. Artificial intelligence-based algorithms that identify predictive factors of response to treatment are already available in cancer and autoimmune disease management [74,75]. In the future, a similar approach that identifies those patients with hemophilia with the lowest risk of complications and the highest expected treatment response from the novel non-replacement drugs will allow clinicians to propose the best treatment option for each patient with a personalized medicine approach. In particular, a registry of real-world data will help identify patient-specific predictive factors of treatment efficacy and safety. In this regard, the ABR is considered an incomplete treatment outcome since it does not fully capture other clinically important outcome domains, such as joint health, treatment adherence, and health-related quality of life [76]. The widely used hemophilia joint health score (HJHS) has become a standard industry outcome measure for licensure trials, although it has been validated only in children. However, physical examination on its own is not a sufficient measure of treatment efficacy [76]. Ultrasound imaging can detect even small amounts of fluid and discriminate between a synovial and a hemorrhagic joint effusion (joint bleeding). Scores, such as the hemophilia early arthropathy detection with ultrasound (HEAD-US) score [77,78], which evaluates synovitis and osteochondral damages that are gaining growing attention for their simple and convenient use in point-of-care ultrasound evaluation of joints during routine and possibly clinical trials. However, these tools still need validation as outcome measures in clinical trials. Both generic and hemophilia-specific health-related quality of life tools are often lengthy and missing data are frequent. As clinical trials are usually conducted under optimal conditions, it is crucial that real-world applicability is demonstrated. This can be difficult if patient-reported outcome measures are not administered as a standard part of routine clinical practice.

Another unsolved clinical problem is the management of patients during trauma and surgery with replacement or bypassing agents to avoid overtreatment and thrombotic risk. As the hemostatic effects of non-replacement drugs cannot be measured using the aPTT or specific factor activity assay, global coagulation assays, although not standardized yet, seem to be the most appropriate techniques, in particular for comparing data in single patients before and after switching to the novel treatment [76]. In the future, studies employing global coagulation tests should be designed to provide standardized information on the hemostatic status of patients receiving these novel therapies.

Another important issue is how to manage patients with inhibitors before and in the course of non-replacement drugs because, to date, it is not clear whether they should undergo immune tolerance induction before switching to these innovative drugs, and with which therapeutic regimen. In addition, in the case of past inhibitors, it is unclear whether periodical administration of FVIII or FIX may help maintain tolerance.

Finally, the occurrence of ADA against these drugs is possible, although not frequent. Laboratories should be equipped with techniques for drug concentration measurement or specific activity assays, and ADA detection assays in any case of suspected loss of efficacy or for periodical monitoring. Alternatively, patient samples should be sent to a reference center with a laboratory with expertise in this activity.

## 5. Materials and Methods

This narrative review was performed by analyzing the medical literature for studies on novel non-replacement drugs for hemophilia treatment. The MEDLINE electronic database was searched without temporal limits, with the English language as a restriction. The following terms were searched for: “newer hemostatic agents”; “non-replacement agents”; “non-replacement drugs”; “novel hemostatic agents”; “investigational drugs”; “hemophilia A”; “hemophilia B”; “inhibitors”; “antithrombotic pathway”; “anticoagulant pathway”; “antithrombin”; “fitusiran”; “serpinPC”; “tissue factor pathway inhibitor (TFPI)”; “concizumab”; “marstacimab”; “befovacimab”; “MG1113”; and “mim8”. We also screened the reference lists of the most relevant review articles for additional studies not found in our initial literature search. Search terms were also used to search for relevant abstracts from the latest international congresses on hematology.

Exclusion criteria were studies on extended half-life factors and emicizumab; animal models and preclinical evidence; studies on acquired hemophilia or other rare bleeding disorders other than hemophilia were excluded from the search.

## 6. Conclusions

In conclusion, novel non-replacement agents will add to the panoply of new treatment options with the potential to change the scenario of hemophilia treatment, raising great expectations among both clinicians and patients with hemophilia.

However, further evidence from completed clinical trials and post-marketing studies is warranted to provide the most effective and safe drug for each patient, in a personalized medicine model that should be the modern paradigm of medicine.

## Figures and Tables

**Table 1:** Thrombotic events reported during clinical trials with novel non-replacement drugs.

Agent	Thromboembolism	Possible Cause and Associated Factor
Fitusiran	**5 events:** 1 cerebral sinus thrombosis 1 atrial thrombosis 1 cerebral infarct 1 cerebrovascular accident 1 spinal artery thrombosis	AT 10–20%, concomitant repeated FVIII, tobacco use AT 10–20%, concomitant repeated FVIIa AT < 10%, recent prostate cancer AT < 10%, story of DVT, diabetes, active smoker AT < 10%, spinal injury, vascular disorder
Anti-TFPI	**3 events** (befovacimab): 1 ischemic stroke 1 retinal artery thrombosis 1 CNS venous thrombosis **5 events** (concizumab): 2 arterial thrombosis 3 venous thrombosis	Dose escalation for all patients, mechanism of action (inhibition of Kunitz-type 1 and 2 domains of TFPI) Presence of baseline thrombotic risk factors, concomitant by-passing agents on the day of the event onset in all patients

AT antithrombin; CNS central nervous system; DVT deep venous thrombosis; FVIIa activated coagulation factor VII; FVIII coagulation factor VIII; TFPI tissue factor pathway inhibitor.

## Data Availability

Data sharing not applicable.
